# Supplementary material for: Nitrous oxide respiring bacteria in biogas digestates for reduced agricultural emissions
Source: ISME J. 2021 Sep 6;16(2):580–90. doi: 10.1038/s41396-021-01101-x (PMC8776835; doi:10.1038/s41396-021-01101-x)
Supplement: Supplementary file 1 — Supplementary Information [file 41396_2021_1101_MOESM1_ESM.docx]

**Supplementary Information**

**N_2_O-respiring bacteria in biogas digestates for reduced agricultural emissions**

Kjell Rune Jonassen, Live H Hagen, Silas HW Vick, Magnus Ø Arntzen, Vincent GH Eijsink, Åsa Frostegård, Pawel Lycus, Lars Molstad, Phillip B Pope, Lars R Bakken

Correspondence to: [lars.bakken@nmbu.no](mailto:lars.bakken@nmbu.no)

This Supplementary Online Material contains 7 sections:

Page

A. Gas kinetics during enrichment culturing 2

B. Growth and decline of members of the methanogenic consortium 9

C. Metabolism of the methanogenic consortium 14

D. Genetics of isolated organisms 18

E. Carbon substrate utilization by isolated organisms 20

F. Denitrification phenotypes of isolated organisms 22

G. Aerobic growth in sterilized digestate, and the effect of the enriched

digestate on N_2_O emissions 35

H. References 41

**A. Gas kinetics during enrichment culturing**

This section provides details of the gas kinetics and modelled bacterial growth for anaerobic enrichment culturing with N_2_O, as well as gas kinetics in additional (control) experiments with other electron acceptors and a different (thermophilic) digestate.


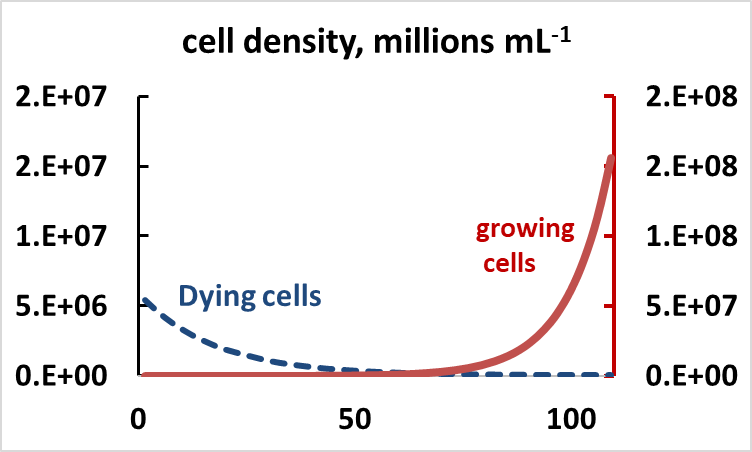


**Figure S1: Modelling growth of** **N_2_O reducing bacteria during enrichment by anaerobic incubation of a mesophilic digestate with N_2_O, based on measured rates of N_2_O reduction to N_2_.**

Panel A shows measured rates of N_2_O reduction to N_2_ (***V_N2_***, µmol N mL^-1^ h^-1^) in the second enrichment culture experiment (Figure 2BC). The values plotted are average of three replicate enrichment vials, with standard deviation as vertical lines. The insert is scaled to visualize the rates during the first 100 h. The fluctuations after 120 hours are due to episodes of N_2_O depletion and subsequent injections of N_2_O (see Figure 2B). Panel B shows the same data plotted on a log scale, illustrating that the rate declined exponentially during the first 30-40 h, and increased exponentially from 60 to 110 h. The kinetics suggested the presence of two groups of N_2_O respiring organisms: One whose respiration died out gradually during the enrichment (**D**), and one which was growing by respiring N_2_O (**G**). To assess the respiration kinetics of **D** and **G**, the following model was used:

$\boldsymbol{V}_{\boldsymbol{N}\boldsymbol{2}}\boldsymbol{=}\boldsymbol{V}_{\boldsymbol{D}}\boldsymbol{+}\boldsymbol{V}_{\boldsymbol{G}}\boldsymbol{=}\boldsymbol{V}_{\boldsymbol{D}\boldsymbol{0}}\boldsymbol{*}\boldsymbol{e}^{\boldsymbol{-dt}}\boldsymbol{+}\boldsymbol{V}_{\boldsymbol{G}\boldsymbol{0}}\boldsymbol{*}\boldsymbol{e}^{\boldsymbol{\mu}\boldsymbol{t}}$

where ***V_D_*** is the rate of N_2_O-reduction (µmol N mL^-1^ h^-1^) by **D**, **V_D0_** is their rate at time zero, and ***d*** is the first order rate of decline (h^-1^); ***V_G_*** is the rate of N_2_O-reduction by **G,** and ***µ*** is the growth rate of **G** (h^-1^). The parameters were estimated by fitting the modeled to the measured data for 0-110 h, using the Generalized Reduced Gradient Solver in Excel, yielding the following results: ***V_D0_***= 30 nmol N mL^-1^h^-1^, ***V_G0_*** = 0.014 nmol N mL^-1^ h^-1^, ***d***= 0.03 h^-1^, ***µ***=0.1 h^-1^. The modelled **V*_N2_*** (black line in panel B) fits very well with the data (r^2^ = 0.997). The rates of N_2_O reduction by the growing and declining groups are shown as red and blue dotted lines. The inserted panel shows the estimated cell densities (millions mL^-1^).

The estimated growth rate of **G** (***µ***=0.1 h^-1^) equals the maximum anaerobic growth rate of the model strain *Paracoccus denitrificans* at 20 ^0^C, as measured by Bergaust et al (2010, 2012), who also determined the growth yield, ***Y*** =1.9*10^13^ cells per mol electrons = 5.9 g cell dryweight per mol electrons (given 310 fg dry-weight per cell).. These parameters were used to estimate the number of “*Paracoccus* equivalent” cells (310 fg dry-weight per cell) in the enrichment culture as shown in Figure 2C in the main paper. For the period 0-110 hours with exponential growth, the cell density was calculated as ***N_G_(t)***= ***V_G_(t)***/***V_e_****_-max_* where ***N_G_(t)*** is the number of cells mL^-1^ at time t (h) after initiation of the enrichment culturing, ***V_G_(t)*** is the rate of N_2_O-reduction (mol N mL^-1^ h^-1^) at time t, and ***V_e_***_-max_ = 5.26*10^-15^ mol e^-^ cell^-1^ h^-1^. The estimated initial number of growing cells (***N_G_(0)***) was 2.7*10^3^ cells mL^-1^, and the number after 110 hours (***N_G_(110)***) was 1.6*10^8^ cells mL^-1^ (numbers are given in Figure 2C in the main paper). After 110 h, the N_2_O-reduction rate ceased to increase exponentially, presumably because the provision of electron donors was insufficient to sustain a growth rate of 0.1 h^-1^ for cell densities >1.6*10^8^ cells mL^-1^. Further growth was thus estimated by ***N_G_(t)***= ***N_G_(110)*** + **∆N_2_(t)*Y**, where ***N_G_(110)*** is the cell density reached at t=110 h (1.6*10^8^mL^-1^), **∆N_2_(t)** is the cumulated of N_2_ produced from 110 h and onwards (mol N mL^-1^), and **Y** is the growth yield = 1.9*10^13^ cells mol^-1^ N. The cell specific rate of electron flow (***V_e-_***, mol e^-^ cell^-1^ h^-1^) throughout the enrichment culturing (Figure 2C of the main paper) was calculated by ***V_e-_(t)***= ***V_N2_(t)/N_G_(t)*** where **V_N2_(t)** is the measured rate of N_2_ production (mol N mL^-1^ h^-1^) and ***N_G_(t)*** is the estimated cell density, both at time t.

**Figure S2: Reproducible N_2_ production rates during anaerobic incubation of a mesophilic digestate with N_2_O.** Several enrichment experiments were run with mesophilic digestates, showing essentially identical N_2_ production kinetics, i.e. declining rates during the first 50 hours, followed by exponential increase during the next 50 h. Enrichment 1 is the experiment used for metagenomics and metaproteomics (Figure 2A, main paper). Enrichment 2 is the experiment shown in Figure 2BC (main paper). All three enrichment experiments were equal except for the different initial concentrations of N_2_O which were 4, 15 and 9 vol% N_2_O in headspace for experiment 1, 2 and 3, respectively. The equilibrium concentrations in the digestate, given the temperature = 20 ^0^C, are 1.1, 4.2 and 2.5 mM N_2_O (Experiment 1, 2 and 3 respectively). In all enrichment experiments there were 3 replicates (vials), and the plotted values are average, with standard deviation as vertical lines. Different initial N_2_O concentrations were used (ranging from 1-4 mM in the digestate; see inserted panel), without any significant effect on the N_2_ kinetics. The insert shows the concentrations of N_2_O in the digestate during the first 100 hours of each enrichment experiment.

**Figure S3: Effects of O_2_, NO_3_^-^ and N_2_O on** **methane production.** Freshly sampled digestate from the mesophilic anaerobic digester (37 °C) was incubated (20 °C) as stirred batches (50 mL in 120 mL vials, three replicates for each treatment), provided with either O_2_, N_2_O, NO_3_^-^, or without any electron acceptors added (=control). Oxygen and N_2_O concentrations were sustained by repeated injections, while NO_3_^-^ was supplied by peristaltic pumping of KNO_3_ via a needle through the septum (1.57M KNO_3_, flow rate 0.02 mL h^-1^ => 31.4 µmol NO_3_^-^ h^-1^). The vials were monitored for gas concentrations (N_2_, NO, N_2_O, O_2_, CH_4_, CO_2_) in the headspace. Panel A shows the methane production rate (V_CH4_) in all treatments (standard deviation shown as vertical lines). While CH_4_ production was effectively suppressed by NO_3_^-^ and N_2_O, V_CH4_ in the vials with O_2_ was 50-75% of that in the control vials (i.e. vials without any electron acceptors, basically resembling the anaerobic digester). The methane production in the mesophilic anaerobic digester was 1.48 µmol CH_4_ mL^-1^ h^-1^ (Materials and Methods), which is an order of magnitude higher than the measured production rate in the control treatment. The temperature difference between the digester and the vials could account for this difference; indeed based on the apparent activation energy (Ea) for methane production in anaerobic digesters, determined by Elsgaard et al (2016) to be 80 kJ mol^-1^, one would predict that the rate declines from 1.48 to 0.24 µmol CH_4_ mL^-1^ h^-1^ by the downshift from 37^o^C (in the digester) to 20 °C (in the vials). This temperature-extrapolated rate of methane production is shown as a dashed line in Panel A. The similarity between the predicted and observed methane production rates in the control vials shows that a competent methanogenic consortium was maintained during culturing at 20 °C. Panel B shows the methane production together with the O_2_ concentration in the headspace (vol % O_2_) for the vials with oxygen in the headspace (standard deviation as vertical lines). The oxygen concentrations in the headspace fluctuated between 0 and 4 vol % (O_2_ injection events marked by red arrows). The low rate of stirring in these experiments (300 rpm), implies relatively slow transport of O_2_ from headspace to the liquid, and probably uneven distribution of O_2_ within the liquid volume. Hence, there may have been anaerobic zones and microsites within the liquid, which could explain the sustained methanogenesis. The coexistence of aerobic and anaerobic metabolism, including methanogenesis is bioreactors has often been observed, and the inhibitory effect of low concentrations of oxygen on the methanogenesis in bioreactors may be marginal (Botheju and Bakke 2011).

**Figure S4:** **Methane production in the enrichment culture used for metagenomics and metaproteomics.** In parallel with the enrichment culturing of N_2_O reducers which was used for -omics analyses **(Figure 2A in the main paper),** we monitored control vials (n=3), i.e. vials without N_2_O in headspace. Panel A shows the N_2_O concentration in the digestate (mM N_2_O), the rates of N_2_ production (***V_N2_***) and methane production (***V_CH4_***) in the vials with N_2_O in the headspace, as well as the rate of methane production in vials without N_2_O (***V_CH4 control_***). Panel B shows cumulated CH_4_ production in the control vials without N_2_O (µmol CH_4_ vial^-1^) and in the vials with N_2_O, as well as the latter expressed as % of CH_4_ accumulation in the control. The data in panel B show that inhibition of methanogenesis by N_2_O was incomplete, the total methane production in the N_2_O enrichment vials being ~10 % of that in the control vials. The insert in panel A shows *V_CH4_*, *V_CH4control_* and *V_N2_* (symbols the same as in the main panel) for the first 90 hours. Hypothetically, the apparent N_2_O inhibition of methanogenesis could be caused by N_2_O-driven methanotrophy (N_2_O replacing O_2_ as co-substrate for methane monooxygenase), but if so, the oxidation of 1 mole CH_4_ would reduce 2 mol of N_2_O to N_2_, i.e. ***V_N2_*** =2*(***V_CH4control_*** -**V_CH4_**). The inserted panel shows that the measured rates of N_2_O-reduction was clearly insufficient, and the hypothesis must be rejected.

**Figure S5: Estimated carbon mineralization with different terminal electron acceptors: O_2_, N_2_O, NO_3_^-^** **and CO_2_**. The Figure shows the effect of O_2_, N_2_O and NO_3_^-^ on apparent C-mineralization rates for the experiment presented in Figure S3. Panels A and C show the estimated C-mineralization for the different pathways (linear and logarithmic scale, in A and C, respectively), based on measured gas consumption/production, and the stoichiometry of the pathways (Panel B). For the vials with NO_3_^-^, the stoichiometry was corrected for the transient accumulation of N_2_O (Panel E), since NO_3_^-^→1/2 N_2_ consumes 5 electrons mol^-1^ N, while NO_3_^-^→1/2 N_2_O consumes only 4. While N_2_O and NO_3_^-^ effectively inhibited methane production, this was not the case for O_2_ (See Figure S3). For this treatment, two curves are shown (Panel A & C): one for the aerobic pathway alone (blue, marked O_2_ in the legend), and one for the sum of aerobic respiration and methanogenesis (marked O_2_+CH_4_ in legend). This shows that aerobic respiration accounts for approximately 50% of the C mineralization.

Panel D: Estimated CO_2_ production based on measure CO_2_ in headspace. The values are uncertain, because the digestate contained large amounts of CO_2_ and HCO_3_^-^ when sampled (high partial pressure of CO_2_ in the digester), and the pH in the digestate was high (7.6), which means that minor changes in pH throughout the incubation would affect the proportion of CO_2_ present as HCO_3_^-^ in the liquid. Nevertheless, the estimated of CO_2_ production showed similar contrasts between treatments as the estimates based on stoichiometry (Panel A): both show a retarded mineralization in the N_2_O treatment during the first 100 h compared to the control and the oxic treatment. No estimates could be made for the treatment with NO_3_^-^ because denitrification raised the pH (measured only at the end), causing declining CO_2_ concentrations in the headspace.

Panel E: Transient accumulation of N_2_O during the incubation with NO_3_^-^. During the first 50 h, NO_3_^-^ was reduced to N_2_O exclusively, reflecting that in the original digestate, bacteria that reduce NO_3_^-^ to N_2_O outnumber those that are able to reduce N_2_O to N_2_. This is corroborated by the estimated C kinetics shown in Panel A-D (early onset of mineralization based on NO_3_^-^ reduction while that based on N_2_O reduction was initially very slow).

**Figure S6: Comparison of N_2_O reducing bacteria in raw sludge and the digestate.** We hypothesized that only a fraction of the N_2_O-reducing bacteria in the sludge would survive the passage through the anaerobic digester, and checked this by investigating the N_2_O reduction kinetics in enrichment cultures with digestate, and digestate which had been heated to 55 ^0^C, with and without the addition of raw sludge (50 mL digestate +/- 1 mL raw sludge in each vial). The panel shows the measured rate of N_2_ production (with standard deviation, n=3) for the four treatments. As in Figure S1, we estimated the initial rate of N_2_O reduction as a proxy for the density of N_2_O-reducing bacteria, using the same model:

$$\boldsymbol{V}_{\boldsymbol{N}\boldsymbol{2}}\boldsymbol{=}\boldsymbol{V}_{\boldsymbol{D}}\boldsymbol{+}\boldsymbol{V}_{\boldsymbol{G}}\boldsymbol{=}\boldsymbol{V}_{\boldsymbol{D}\boldsymbol{0}}\boldsymbol{*}\boldsymbol{e}^{\boldsymbol{-dt}}\boldsymbol{+}\boldsymbol{V}_{\boldsymbol{G}\boldsymbol{0}}\boldsymbol{*}\boldsymbol{e}^{\boldsymbol{\mu}\boldsymbol{t}}$$

The model was fitted to the data for each single vial, using the Generalized Reduced Gradient Solver in Excel, resulting in three independent estimates of ***V_G0_*** for each treatment (one for each replicate vial). Panel A shows measured ***V_N2_*** for all treatments plotted against time (average values, standard deviation as vertical lines, n=3). The boxes show the average estimated initial rates ***V_G0_*** as µmol N vial^-1^ h^-1^, with standard deviations in parenthesis (n=3). Panel B shows the same data with a log scaled Y-axis.

***V_G0_*** in the 1 mL sludge added can be estimated by the increase in ***V_G0_*** by adding 1 mL sludge to the digestates:

Unheated digestate: **V_G0_sludge_** = ***V_G0_digestate+sludge_*** – **V*_G0-digestate_*** = 0.15 µmol N mL^-1^ sludge h^-1^

Heated digestate: **V_G0_sludge_** = ***V_G0_digestate55+sludge_*** – **V*_G0-digestate55_*** = 0.81 µmol N mL^-1^ sludge h^-1^

The two **V_G0_sludge_** estimates are very different, but they are both much higher than **V*_G0-digestate_***, which was 2.67 µmol N vial^-1^ h^-1^ (panel A) = 0.05 µmol N mL^-1^ digestate h^-1^. The fraction of N_2_O reducers which survives the passage of the anaerobic digester is estimated by **F=** **V*_G0-digestate_*/V_G0_sludge_**, and F= 0.33 and 0.06 (based on the two widely different estimates of **V_G0_sludge_**. A reasonable conclusion is that ≤1/3 of the viable N_2_O-reducing organisms in the sludge survived the anaerobic digestion.

**Figure S7: Comparison of digestates from a mesophilic and a thermophilic digester.**  We sampled digestates from the mesophilic and thermophilic digesters (37 °C and 52 °C respectively, both fed with the same sewage sludge), and incubated them anaerobically with N_2_O at 20 ^o^C as in previously presented experiments (**Figure 2** main paper, **Figures S1-5**). Measurements of N_2_ production (this Figure) showed that the thermophilic digestate contained orders of magnitude lower number of N_2_O-respiring organisms than the mesophilic digestate.

**B. Growth and decline of members of the microbial consortium**

This section provides data regarding the growth or decline of different members of the microbial consortium of the digestate based on the abundance of individual MAGs in the metagenomes (**Supplementary Data S2)** and metaproteomes (**Supplementary Data S1)** at three timepoints during the enrichment (0, 115 and 325 hours; see Figure 2 and 3 in main paper).

**Figure S8:** **Abundance of MAGs during enrichment culturing.** To assess how enrichment culturing with N_2_O affected the abundance of members of the microbial consortium, we used the metagenomic and metagenomic data to calculate the relative increase of each MAG: $S=\frac{q}{\overline{x}}$ , where *q* is the regression coefficient for *x* (x=coverage for genomics, sum of LFQ for proteomics) against time and $\overline{x}$ is the average for the MAG (all three time points), thus the unit for S is h^-1^. The plot shows *S* from proteomics against *S* based on genomics for each MAG. MAGs with *nosZ* are marked with red circles. For the majority of MAGs, S ranged from -0.005 to +0.005 h^-1^, regardless of the assessment method. The identity of the MAGs is shown for MAGS with at least one S-value outside this range. Only two of the MAGs with *nosZ* had S > 0.005 h^-1^ (MAG260 & MAG268). For the organisms without *nosZ*, the average S was -0.0013 h^-1^ for metagenomics data and -0.00045 h^-1^ for metaproteomics data.

**Figure S9: Evaluation of growth/decline of MAGs without *nosZ*, stratified according to relative abundance.** To inspect if the growth/decline differed depending on the initial population size, we stratified the MAGs into three groups, i.e. MAGs with initial genomic abundance > 0.5 %, 0.1-0.5 % and < 0.1 %, and plotted the relative increase, *S* (as calculated for Figure S8) as calculated from proteomics against that from genomics. Panel A shows the plot of for all MAGS, and Panels C-D shows the plots for the three strata. The stratification demonstrated no clear relationship between initial abundance and the apparent ability to survive during the enrichment culturing: within each group, the majority of MAGs clustered around zero, while a minority showed a declining trend, both for the genomics and the proteomics.

**Figure S10: Apparent rate of growth/decline of MAGs by combining -omics and measured abundance of 16SrDNA.** As a final approach to evaluate growth and decline of specific populations during the enrichment culturing, we combined omics data with 16SrRNA gene abundance (16S copies mL^-1^ digestate measured by digital droplet PCR, using universal primers), to assess the abundance of individual MAGs and to calculate the apparent growth rates (or decline) during enrichment culturing. Average total 16S rRNA gene abundance at each time point is shown in the inserted in panel, with standard error (n=3). For each time point (t= 0, 115 and 325 h), the cell density of each MAG was assessed by ***N_it_=S_t_*C_it_/∑C_t_*** where ***S_t_*** is the measured 16SrDNA abundance at time ***t***, and ***C_it_******/∑C_t_*** is the MAG’s relative abundance at time t. For genomics, ***C_it_*** = the MAG’s coverage at time t, ***∑C_t_*** = the total read coverage of all 149 MAGS at time. For proteomics, ***C_it_*** = the pooled LFQ value for the MAG at time t, ***∑C_t_*** = the sum of pooled LFQ for all MAGS at time t. The apparent growth/death rate was estimated by the slope of **ln(N_it_)** against time (linear regression). The results indicate slight growth (0-0.005 h^-1^) for the majority of organisms (upper right quadrant), consistent strong growth for *nosZ* encoding MAG260 and MAG268, and consistent decline for 9 MAGs (lower left quadrant). The genomics- and proteomics-based µ were inconsistent for 8 MAGs (upper left and lower right quadrant). The relative abundance of the 9 declining MAGs is listed in **Table S1**). We did not find any convincing common traits between the MAGs that could explain their decline. One possible reason for their declining abundance could be inability to adapt to the lower temperature (20^o^C in the enrichment culture versus 37^o^C in the digester).

**Table S1: Relative abundance from % total read coverage and % LFQ for MAGs that declined during the enrichment.** GTDB classifications were assigned at a phylum level. MAG relative abundance was calculated as % of total read coverage of the 149 MAGs and from relative LFQ%, which denotes the relative protein intensity calculated as % of LFQ assigned to an individual MAG relative to the summed LFQ for the 149 MAGs used to construct the metaproteome database.

| **MAG ID** | **GTDB classification (phylum)** | **Relative abundance (%)** | | | **Relative LFQ (%)** | | |
| --- | --- | --- | --- | --- | --- | --- | --- |
|  |  | **Time (h)** | | | **Time (h)** | | |
|  |  | **0** | **115** | **325** | **0** | **115** | **325** |
| MAG20 | Myxococcota | 0.764 | 0.176 | 0.001 | 4.076 | 1.017 | 0.388 |
| MAG13 | Spirochaetota | 1.105 | 0.248 | 0.052 | 0.915 | 0.213 | 0.209 |
| MAG132 | Spirochaetota | 0.082 | 0.004 | 0.001 | 0.050 | 0.026 | 0.016 |
| MAG30 | Spirochaetota | 0.511 | 0.144 | 0.033 | 2.895 | 1.155 | 0.872 |
| MAG58 | Spirochaetota | 0.269 | 0.015 | 0.005 | 1.933 | 0.459 | 0.330 |
| MAG33 | Desulfobacterota | 0.531 | 0.264 | 0.020 | 0.339 | 0.176 | 0.064 |
| MAG125 | Firmicutes | 0.104 | 0.031 | 0.005 | 0.510 | 0.166 | 0.056 |
| MAG115 | Firmicutes | 0.108 | 0.097 | 0.025 | 0.135 | 0.096 | 0.021 |
| MAG118 | Thermotogota | 0.105 | 0.055 | 0.016 | 1.386 | 0.601 | 0.141 |

**Figure S11: Abundance of MAGs with *nosZ***. Panel A shows the relative abundance of the *nosZ* containing MAGs based on genomics (reads as fraction of the sum of 149 MAGs). Panel B shows the relative abundance based on proteomics (LFQ as fraction of the sum of 149 MAGs). Panel C shows a crude estimate of apparent growth rates based on genomics and proteomics (= slope of ln(N) against time; N=relative abundance). These results demonstrate substantial growth for MAG260 and MAG268, but not for the other MAGs. In panel D, the sum of MAG260 and 268 is plotted against time, together with cumulated N_2_ (derived from data shown in Figure 2, main paper). The inserted panel shows the same data on a log scale. This shows that the sum of the abundance of the two MAGs (based on proteomics of genomics) increased as cumulated N_2_O-reduction to N_2_ increased.

**C. Metabolism of the methanogenic consortium**

Here we present a metaproteome-centric metabolic map of possible substrate flows in the microbial consortium, and experimental evidence for the predicted effects of N_2_O-inhibition of methanogenesis in this consortium: accumulation of volatile fatty acids and hydrogen.


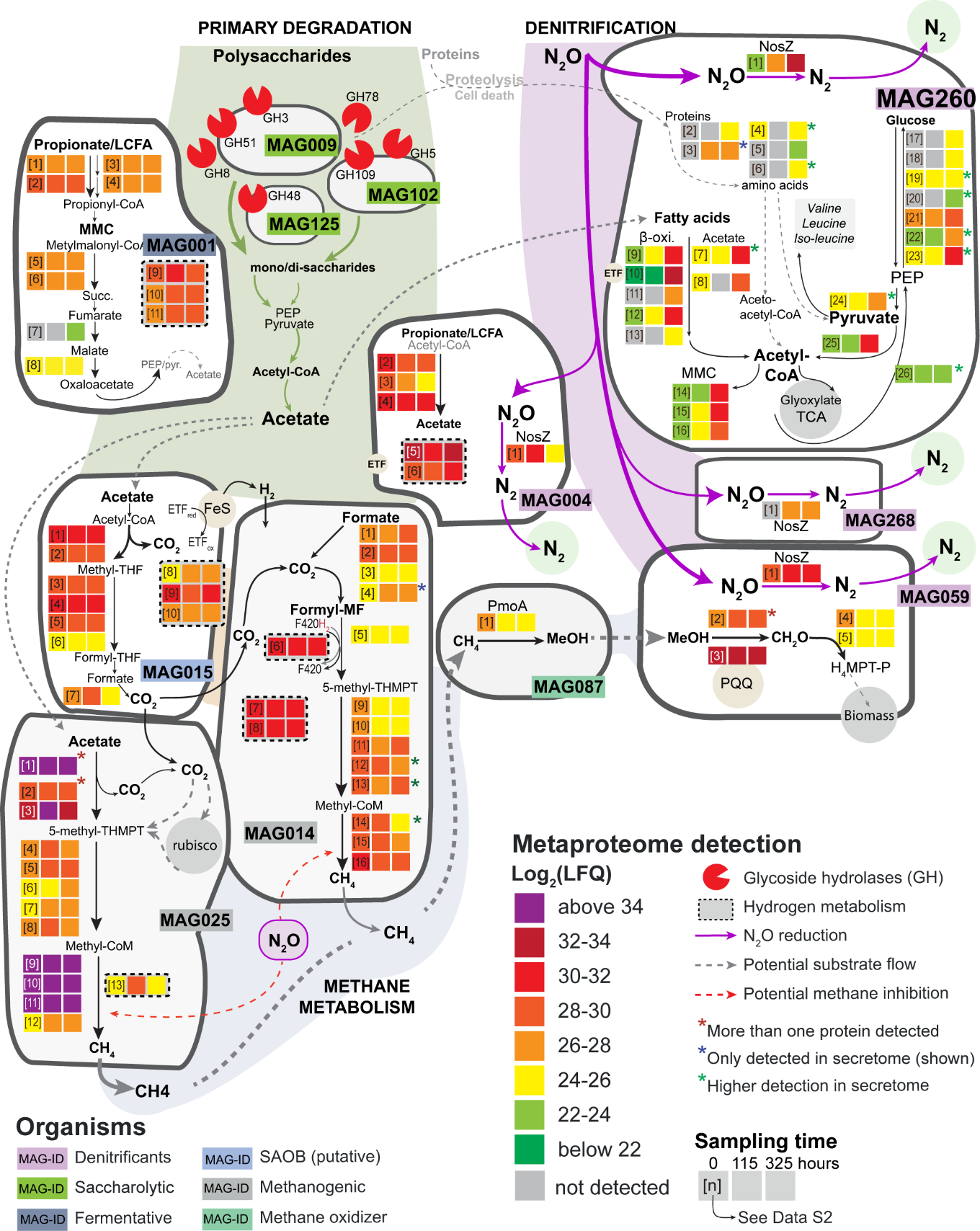


**Figure S12: Metaproteome-centric metabolic map of the substrate flow in the microbial consortium.** For metabolic reconstruction of the substrate flow, including primary degradation of carbon sources and N_2_O reduction, we scanned the detected proteins affiliated to each MAG for enzymes involved in specific metabolic pathways. Detected protein levels (log_2_(LFQ)) for the three sampling timepoints (after 0, 115 and 325 hours) are indicated by colored squares, where the number in the first square corresponds to the number in the first column in **Supplementary Data S2**. A variety of carbohydrate-active enzymes (CAZymes), including those active on cellulose (members of GH5, GH8 and GH48) were detected in the metaproteome. Only a selection is shown in the figure; a complete list of detected proteins annotated as CAZymes can also be found in **Supplementary Data S2**. Multiple MAGs also expressed proteins for fermentation processes and production of acetate and propionate. The metaproteome further supported the assumption that these fermentation products are metabolized by a population which included N_2_O-reducers, represented by MAG004 and MAG260 in the figure. N_2_O has been suggested to inhibit the enzymatic process of methanogenesis (Andalib et al 2011, Kengen et al 1988), which was supported by reduced methane-production rate in the microbial enrichment incubated with N_2_O in the current study. Yet, the protein detection level of key enzymes involved in both hydrogenotrophic and acetoclastic methanogenesis (MAG014 and MAG025, respectively) were amongst the highest detected in the metaproteome, even after 115 and 325 hours of incubation. Another MAG with numerous highly detected proteins was affiliated to *Dethiobacteria*, a class recently suggested to encompass syntrophic acetate oxidizing bacteria (SAOBs) (Mosbæk et al 2016, Dyksma et al 2020). This MAG, MAG015, expressed proteins related to the Wood-Ljungdahl pathway (WLP), and combined with the detection of an electron transfer complex (gene cluster encompassing iron-sulfur ferredoxin, coenzyme F_420_ hydrogenase/dehydrogenase, electron transfer flavoprotein) and an aldehyde ferredoxin oxidoreductase for potential acetate activation (Swanson et al 2008, Keller et al 2019) we postulate that also this representative of *Dethiobacteria* might use WLP in a reverse direction to oxidize acetate. This was strengthened by the detection of enzymes central for β-oxidation of longer-chained fatty acids and the detection of the fructose diphosphatase used in anabolic metabolism (i.e., gluconeogenesis). Importantly, SAOBs are depending on an active hydrogen-scavenger population, often hydrogenotrophic methanogens (such as MAG014), to realize the oxidative direction of WLP, which reinforces our observations that the consortium is synergistically producing methane at some capacity. Finally, we detected predicted methane monooxygenase and methanol dehydrogenase proteins from MAG087 and MAG059 (respectively), which leaves tantalizing hypotheses as to the potential role of the methanotrophic community within this enrichment. Methanotrophic processes have recently been shown to be facilitated by the presence of N_2_O as a terminal electron acceptor (Valenzuela et al 2020; Cheng et al 2019), but direct links, which may possibly exist within this enrichment, remain to be elucidated.

**A**

**Figure S13: Quantification of volatile fatty acids (VFA) and H_2_ during enrichment culturing**. Inhibition of methanogenesis by N_2_O could result in transient accumulation of intermediates such as VFA (**Figure S12**), which might last until the N_2_O-respiring bacteria have become sufficiently numerous to effectively reap these intermediates. H_2_ might also accumulate, until the partial pressure of H_2_ reaches levels high enough to sustain hydrogenotrophic acetogenesis (Wood-Ljungdahl pathway).

**B**

Panel A shows the VFA concentrations (mmol L^-1^) in samples of digestate directly from the digester (Digester, n=3 replicates, frozen immediately after sampling from the anaerobic digester), and at the three time points (0, 115 and 325 h) of the enrichment culturing experiment number 1, presented in **Figure 2A** in the main paper (n=2 for t=115 h, and 3 for the others). All samples were stored at -80 °C before being prepared for VFA analysis. The lower concentration at the onset of the enrichment culturing (t=0) compared to that in the digester could be due to oxygenation during transport from the WWTP to the laboratory, and to losses due to the He-washing (evacuation and He-filling) prior to enrichment culturing. The results clearly show the expected transient accumulation of VFAs.

Panel B shows the accumulation of H_2_ in response to N_2_O-mediated inhibition of methane production. This was measured in a repetition of the enrichment culturing shown in **Figure 2** of the main paper, using an improved version of the incubation robot system which measures H_2_ by a Plasma Emission detector (PED) (©LDetek). The left panel shows the results for vials with N_2_O in the headspace: concentration of N_2_O in the liquid, concentration of H_2_ in the headspace, and the rate of CH_4_ production (V_CH4_). The insert is scaled to show the onset of CH_4_ production in response to N_2_O depletion. The right panel shows H_2_ and V_CH4_ in vials without N_2_O. These results corroborate the hypothesis that H_2_ accumulates in response to N_2_O-inhibition of methanogenesis, reaching an apparent steady state concentration around 350 ppm in the headspace (P_H2_=3.5*10^-4^ bar = 0.28 µM H_2_ in the liquid).

**
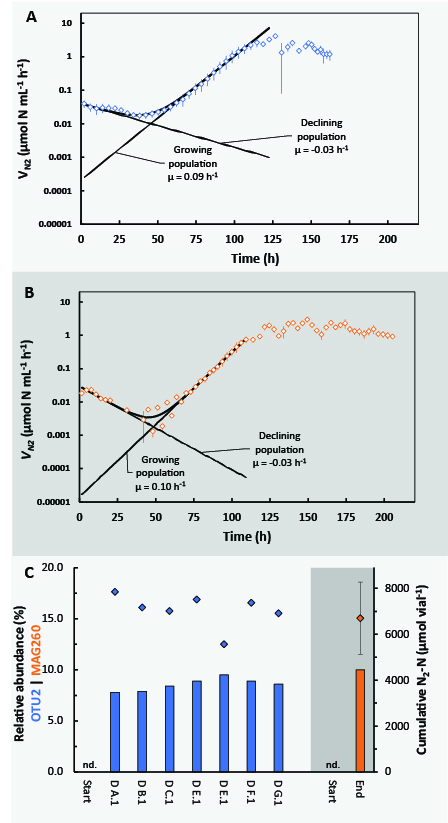
Figure S14 Replication of the enrichment culturing, analysed by** **16RrRNA gene amplicon sequencing.**

A forthcoming paper (preprint on BiorXiv: Jonassen et al 2021) includes 7 parallel enrichment cultures analysed by 16RrRNA gene amplicon sequencing at the end of the incubation. The results confirm the reproducibility of the gas kinetics as well as the enrichment of organisms circumscribed by MAG 260: the Operational Taxonomic Unit no 2 (OTU2) reached dominance in all the 7 cultures. The 16S sequence obtained from the Azonexus sp. had 98.2 % sequence similarity with the overlapping region of the OTU2 consensus sequence, and had 99.2 % sequence identity in the overlapping region when comparing the sequence to the full length 16SrRNA gene of the isolate *Azonexus sp.*

Panel A**:** N_2_-N production kinetics (Error bars = standard deviation, n = 7). Fat black line: modelled N_2_-N production rates based on a growing and dying population of N_2_O reducing bacteria (stippled lines show the modelled activity of the two populations). Panel B**:** a replica of Figure S1B, for comparison. Panel C**:** cumulated N_2_-production (blue diamonds) and relative abundance of OTU2 (blue bars) in the 7 replicated enrichment cultures (D_A.1_ – D_G.1_ ), compared with the cumulated N_2_-production (orange diamond) and the relative abundance of MAG260 (orange bar) in the present experiments.

**D. Genetics of isolated organisms**

Here, we present the results of genome sequencing of the three isolates, their phylogeny, their core denitrification reductase genes as well as genes coding for peripheral proteins which contributes to the denitrification pathway.

**Fig S15: Phylogeny and denitrification genes annotated in draft genomes of isolated organisms**. The panel shows the maximum likelihood phylogenetic trees of the three isolates, based on full length 16S rRNA DNA sequences (bootstrap values > 0.6 , 100 resamplings), their core denitrification genes coding for the four denitrification reductases (Nar/Nap, Nir, Nor and Nos), as well as a number of genes coding for peripheral proteins that contribute to a fully functional denitrification pathway (Vaccaro et al 2016). One of these is *nosR,* which was only found in *Pseudomonas* *sp*. PS. NosR is hypothesized to be involved in electron donation to Nos (Wunsch and Zumft 2005; Zhang et al 2017), but apparently only to Nos Clade I, because organisms with *nosZ clade II* often lack *nosR* (Hein et al 2017), which was the case for the two isolates with nosZ Clade II (AS and AN). *Pseudomonas* *sp*. PS lacked *nosX*, a flavin donor involved in maturation of norR, but insteadthe *apbE* (coding for flavin transferase, EC: 2.7.1.180) that has been suggested as a flavin donor candidate in maturation via covalent flavinylation of NosR in *Pseudomonas stutzeri* (Zhang et al 2017).

**Table S2**: QUAST quality parameters, PROKKA annotation summary, CheckM genome quality parameters and coverage of SPAdes assembled contigs of *Pseudomonas* *sp*. PS, *Azospira* *sp*. AS and *Azonexus* *sp*. AN.

|  | *Pseudomonas* *sp.* PS | *Azospira* *sp.* AS | *Azonexus* *sp.* AN |
| --- | --- | --- | --- |
|  | QUAST quality parameters: | | |
| Contigs total: | 21 | 75 | 59 |
| Largest contig (bp): | 778 581 | 409 855 | 279 444 |
| Contigs (>= 0 bp): | 21 | 75 | 59 |
| Contigs (>= 1000 bp) | 18 | 68 | 48 |
| Contigs (>= 10 000 bp) | 13 | 44 | 26 |
| Contigs (>= 100 000 bp) | 9 | 11 | 13 |
| Total length (bp): | 3 378 613 | 3 810 942 | 2 882 318 |
| N50: | 440 975 | 133 847 | 176 807 |
| L50: | 3 | 9 | 7 |
| Predicted genes (>= 300 bp): | 2349 | 3019 + 11 partially | 2364 + 6 partially. |
| GC (%): | 47.89 | 65.42 | 60.82 |
| Mismatches: |  |  |  |
| # N’s | 195 | 199 | 109 |
| # N’s per 100 kbp | 5.77 | 5.22 | 3.78 |
|  | SPAdes output | | |
| Coverage (k-mer): | 163.8x | 59.72x | 90.12x |
|  | Prokka annotation summary: | | |
| Number of genes predicted: | 3153 | 3518 | 2766 |
| Number of protein coding genes: | 3104 | 3461 | 2713 |
| Number of genes with non-hypothetical function: | 2201 | 2404 | 1839 |
| Number of genes with EC-number: | 1223 | 1247 | 988 |
| Number of genes with Seed Subsystem Ontology: | 972 | 995 | 807 |
| Average protein length: | 323 | 327 | 319 |
|  | CheckM genome quality parameters: | | |
| Marker Lineage | C_Gammaproteobacteria | C_Betaproteobacteria | C_Betaproteobacteria |
| #Genomes | 263 | 223 | 233 |
| #Markers | 507 | 424 | 425 |
| #Marker sets | 232 | 211 | 211 |
| 0 | 6 | 0 | 4 |
| 1 | 498 | 422 | 421 |
| 2 | 3 | 2 | 0 |
| 3 | 0 | 0 | 0 |
| 4 | 0 | 0 | 0 |
| 5+ | 0 | 0 | 0 |
| Completeness | 97.7 | 100.0 | 98.1 |
| Contamination | 0.89 | 0.26 | 0.00 |

**E. Carbon substrate utilization by isolated organisms**

Here we show the results of the testing C substrate utilization by the three isolated organisms. The result show that PS (P*seudomonas sp*.) could utilize a wide specter of substrates, although it’s capacity to utilize polymers was marginal. In contrast AN (*Azonexus sp.*) and AS (*Azospira sp.*) utilized very few C substrates, primarily intermediates of anaerobic fermentation of a methanogenic consortium.

**Table S3:** **Screening the three isolates for C utilization** using Biolog Phenotype MicroArray plates PM1 and PM2, which tests the isolates capacity to utilize various C substrates. Positive wells are marked with + (n=3 replicate plates)

| **PM1** | **PS** | **AS** | **AN** | **PM2** | PS | AS | AN |
| --- | --- | --- | --- | --- | --- | --- | --- |
| A2 L-Arabinose |  |  |  | A2 Chondroitin Sulfate C |  |  |  |
| A3 N-Acetyl-DGlucosamine | **+++** |  |  | A3 α-Cyclodextrin |  |  |  |
| A4 D-Saccharic Acid |  |  |  | A4 ß-Cyclodextrin |  |  |  |
| A5 Succinic Acid | **+++** | **+++** | **+++** | A5 γ-Cyclodextrin |  |  |  |
| A6 D-Galactose |  |  |  | A6 Dextrin |  |  |  |
| A7 L-Aspartic Acid | **+++** | **+++** |  | A7 Gelatin |  |  |  |
| A8 L-Proline | **+++** |  |  | A8 Glycogen |  |  |  |
| A9 D-Alanine | **+++** |  |  | A9 Inulin |  |  |  |
| A10 D-Trehalose |  |  |  | A10 Laminarin | **+++** |  |  |
| A11 D-Mannose | **+++** |  |  | A11 Mannan |  |  |  |
| A12 Dulcitol |  |  |  | A12 Pectin |  |  |  |
| B1 D-Serine |  |  |  | B1 N-Acetyl-DGalactosamine |  |  |  |
| B2 D-Sorbitol |  |  |  | B2 N-AcetylNeuraminic Acid |  |  |  |
| B3 Glycerol | **+++** |  |  | B3 ß-D-Allose |  |  |  |
| B4 L-Fucose |  |  |  | B4 Amygdalin |  |  |  |
| B5 D-Glucuronic Acid |  |  |  | B5 D-Arabinose |  |  |  |
| B6 D-Gluconic Acid | **+++** |  |  | B6 D-Arabitol |  |  |  |
| B7 D,L-α-GlycerolPhosphate | **+++** |  |  | B7 L-Arabitol | **+++** |  |  |
| B8 D-Xylose |  |  |  | B8 Arbutin |  |  |  |
| B9 L-Lactic Acid | **+++** | **+++** |  | B9 2-Deoxy-DRibose | **+++** |  |  |
| B10 Formic Acid |  |  |  | B10 i-Erythritol |  |  |  |
| B11 D-Mannitol |  |  |  | B11 D-Fucose |  |  |  |
| B12 L-Glutamic Acid | **+++** | **+++** | **+++** | B12 3-0-ß-DGalactopyranosylD-Arabinose |  |  |  |
| C1 D-Glucose-6- Phosphate | **+++** |  |  | C1 Gentiobiose |  |  |  |
| C2 D-Galactonic Acid-γ-Lactone |  |  |  | C2 L-Glucose |  |  |  |
| C3 D,L-Malic Acid | **+++** | **+++** | **+++** | C3 Lactitol |  |  |  |
| C4 D-Ribose | **+++** |  |  | C4 D-Melezitose |  |  |  |
| C5 Tween 20 |  |  |  | C5 Maltitol |  |  |  |
| C6 L-Rhamnose |  |  |  | C6 α-Methyl-DGlucoside |  |  |  |
| C7 D-Fructose | **+++** |  |  | C7 ß-Methyl-DGalactoside |  |  |  |
| C8 Acetic Acid | **+++** | **+++** | **+++** | C8 3-Methyl Glucose |  |  |  |
| C9 α-D-Glucose | **+++** |  |  | C9 ß-Methyl-DGlucuronic Acid |  |  |  |
| C10 Maltose |  |  |  | C10 α-Methyl-DMannoside |  |  |  |
| C11 D-Melibiose |  |  |  | C11 ß-Methyl-DXyloside |  |  |  |
| C12 Thymidine | **+++** |  |  | C12 Palatinose |  |  |  |
| D1 L-Asparagine | **+++** |  |  | D1 D-Raffinose |  |  |  |
| D2 D-Aspartic Acid |  |  |  | D2 Salicin |  |  |  |
| D3 D-Glucosaminic Acid |  |  |  | D3 Sedoheptulosan |  |  |  |
| D4 1,2-Propanediol |  |  |  | D4 L-Sorbose |  |  |  |
| D5 Tween 40 |  |  |  | D5 Stachyose |  |  |  |
| D6 α-Keto-Glutaric Acid | **+++** | **+++** |  | D6 D-Tagatose |  |  |  |
| D7 α-Keto-Butyric Acid |  |  |  | D7 Turanose |  |  |  |
| D8 α-Methyl-DGalactoside |  |  |  | D8 Xylitol |  |  |  |
| D9 α-D-Lactose |  |  |  | D9 N-Acetyl-DGlucosaminitol |  |  |  |
| D10 Lactulose |  |  |  | D10 γ-Amino Butyric Acid |  |  |  |
| D11 Sucrose |  |  |  | D11 δ-Amino Valeric Acid |  |  |  |
| D12 Uridine | **+++** |  |  | D12 Butyric Acid |  | **+++** | **+++** |
| E1 L-Glutamine | **+++** |  |  | E1 Capric Acid | **+++** |  |  |
| E2 m-Tartaric Acid |  |  |  | E2 Caproic Acid | **+++** | **+++** |  |
| E3 D-Glucose-1- Phosphate | **+++** |  |  | E3 Citraconic Acid |  |  |  |
| E4 D-Fructose-6- Phosphate | **+++** |  |  | E4 Citramalic Acid |  |  |  |
| E5 Tween 80 |  |  |  | E5 D-Glucosamine | **+++** |  |  |
| E6 α-Hydroxy Glutaric Acid-γLactone |  |  |  | E6 2-Hydroxy Benzoic Acid |  |  |  |
| E7 α-Hydroxy Butyric Acid | **+++** |  |  | E7 4-Hydroxy Benzoic Acid |  |  |  |
| E8 ß-Methyl-DGlucoside |  |  |  | E8 ß-Hydroxy Butyric Acid |  | **+++** | **+++** |
| E9 Adonitol | **+++** |  |  | E9 Glycolic Acid |  |  |  |
| E10 Maltotriose |  |  |  | E10 α-Keto-Valeric Acid |  |  |  |
| E11 2-Deoxy Adenosine | **+++** |  |  | E11 Itaconic Acid |  |  |  |
| E12 Adenosine | **+++** |  |  | E12 5-Keto-DGluconic Acid |  |  |  |
| F1 Glycyl-L-Aspartic Acid | **+++** |  |  | F1 D-Lactic Acid Methyl Ester |  |  |  |
| F2 Citric Acid | **+++** |  |  | F2 Malonic Acid |  |  |  |
| F3 myo-Inositol |  |  |  | F3 Melibionic Acid |  |  |  |
| F4 D-Threonine |  |  |  | F4 Oxalic Acid |  |  |  |
| F5 Fumaric Acid | **+++** | **+++** | **+++** | F5 Oxalomalic Acid |  |  |  |
| F6 Bromo Succinic Acid | **+++** | **+++** | **+++** | F6 Quinic Acid |  |  |  |
| F7 Propionic Acid | **+++** | **+++** |  | F7 D-Ribono-1,4- Lactone |  |  |  |
| F8 Mucic Acid |  |  |  | F8 Sebacic Acid |  |  |  |
| F9 Glycolic Acid |  |  |  | F9 Sorbic Acid |  |  |  |
| F10 Glyoxylic Acid |  |  |  | F10 Succinamic Acid |  |  |  |
| F11 D-Cellobiose |  |  |  | F11 D-Tartaric Acid |  | **+++** |  |
| F12 Inosine | **+++** |  |  | F12 L-Tartaric Acid |  |  |  |
| G1 Glycyl-LGlutamic Acid | **+++** |  |  | G1 Acetamide |  |  |  |
| G2 Tricarballylic Acid |  |  |  | G2 L-Alaninamide |  |  |  |
| G3 L-Serine | **+++** |  |  | G3 N-Acetyl-LGlutamic Acid |  |  |  |
| G4 L-Threonine | **+++** |  |  | G4 L-Arginine | **+++** |  |  |
| G5 L-Alanine | **+++** |  |  | G5 Glycine |  |  |  |
| G6 L-Alanyl-Glycine | **+++** |  |  | G6 L-Histidine | **+++** |  |  |
| G7 Acetoacetic Acid | **+++** |  |  | G7 L-Homoserine |  |  |  |
| G8 N-Acetyl-ß-DMannosamine |  |  |  | G8 Hydroxy-LProline | **+++** |  |  |
| G9 Mono Methyl Succinate |  |  |  | G9 L-Isoleucine |  |  |  |
| G10 Methyl Pyruvate | **+++** | **+++** |  | G10 L-Leucine | **+++** |  |  |
| G11 D-Malic Acid | **+++** |  |  | G11 L-Lysine G |  |  |  |
| G12 L-Malic Acid | **+++** | **+++** | **+++** | 12 L-Methionine | **+++** |  |  |
| H1 Glycyl-L-Proline | **+++** |  |  | H1 L-Ornithine |  |  |  |
| H2 p-Hydroxy Phenyl Acetic Acid | **+++** |  |  | H2 L-Phenylalanine | **+++** |  |  |
| H3 m-Hydroxy Phenyl Acetic Acid | **+++** |  |  | H3 L-Pyroglutamic Acid | **+++** |  |  |
| H4 Tyramine | **+++** |  |  | H4 L-Valine |  |  |  |
| H5 D-Psicose |  |  |  | H5 D,L-Carnitine |  |  |  |
| H6 L-Lyxose |  |  |  | H6 Sec-Butylamine |  |  |  |
| H7 Glucuronamide |  |  |  | H7 D,L-Octopamine |  |  |  |
| H8 Pyruvic Acid | **+++** | **+++** |  | H8 Putrescine |  |  |  |
| H9 L-Galactonic Acid-γ-Lactone |  |  |  | H9 Dihydroxy Acetone |  |  |  |
| H10 D-Galacturonic Acid |  |  |  | H10 2,3-Butanediol |  |  |  |
| H11 Phenylethylamine |  |  |  | H11 2,3-Butanedione |  |  |  |
| H12 2-Aminoethanol |  |  |  | H12 3-Hydroxy-2- Butanone |  |  |  |

**F. Denitrification phenotypes of isolated organisms**

Here we present a series of experiments with each of the three isolated organisms, designed to characterize their denitrification regulatory phenotype, with emphasis on regulatory traits that could determine their capacity to function as sinks for N_2_O in soil. The section starts with a synopsis of the results, with references to the subsequent figures showing the results of individual experiments.

In these experiments, cells were raised under strict aerobic conditions to secure negligible amounts of denitrification reductases in the cells. They were then inoculated to 120 mL vials with He + ~1vol% O_2_ (with or without N_2_O) in the headspace, containing 50 mL of Sistrom’s succinate medium, either with NO_3_^-^ or NO_2_^-^ (1 mM), and with a Teflon-coated magnetic bars). The vials were placed in the thermostatic water bath (20 ^o^C) of the incubation robot, stirred continuously at high speed (700 rpm), and monitored for gas kinetics (O_2_, NO, N_2_O and N_2_) by frequent sampling of the headspace as the culture grows by aerobic respiration, depletes the oxygen and is forced to switch to denitrification. For each gas sample withdrawn, an equal volume of He is returned, and this dilution by sampling is taken into account when estimating the rates of gas production/consumption. Miniscule leakage of N_2_ during sampling (40 -100 nmol) is also taken into account. In addition to the automatized gas sampling, small liquid volumes 20-100 µL were withdrawn manually (syringe) for determining the concentration of NO_2_^-^. The measured concentration of each gas in the headspace is used to calculate its concentration in the liquid, and the molar amount per vial (see Molstad et al 2007).

Each experiment is normally continued until metabolism comes to a halt due to depletion of all electron acceptors, i.e. that the only N-gas present is N_2_, and that the production of N_2_ comes to a halt (cumulative N_2_ reach a stable plateau). NB: cumulative N_2_ is total amount of N_2_ produced at any time *t* is *N_t_* = *N_t_* -*N_0_*+*SN_t_*-*LN_t_*, where *N_t_* is measured amount of N_2_ (vial^-1^) at time *t*, *N_0_* is the measured initial N_2_ in the vial, *SN_t_* is the amount of N_2_ removed by all samplings prior to *t* and *LN_t_* is the amount of N_2_ leaked into the vial prior to sampling at time *t*.

Since the initial concentration of NO_3_^-^, NO_2_^-^ and N_2_O in each vial is known, and that all N-gases (NO, N_2_O and N_2_) are quantified, N-mass balance can be calculated throughout each experiment. For such mass balance, the sampling loss of N_2_O and NO is also taken into account). This is useful for two purposes: 1: to check if the initial amounts of N_2_O+NO_3_^-^+NO_2_-N is recovered as N_2_-N at the end, i.e. when cumulated N_2_ reach a plateau, 2: to estimate the concentration of NO_3_^-^ (or NO_2_^-^) throughout the incubation by mass balance calculation. For obvious reasons 2) can only be done with confidence if 100 % conversion to N_2_ is confirmed, which was the case for all experiments (+/- 5%, ascribed to experimental error).

The convention when reporting the results is to express the amounts of each N-species as molar amounts if N per vial (2 mol N per mol N_2_O and N_2_!), to make the presentations more transparent with respect to N mass balance (1 mol NO_3_^-^ is converted to 0.5 mol N_2_, but 2 mol N_2_-N). The concentrations in the liquid are reported conventionally however (ex: 1 nM N_2_O is 1 nmol N_2_O L^-1^).

The elaborated routines for calculating rates of production/consumption of each gas has been explained in detail by Molstad et al (2007), and the excel program is freely available (Bakken 2020)

**Synopsis of the results**

***Azonexus* *sp*. (AN) Fig S16-18. AN** reduced NO_3_^-^ quantitatively to N_2_, with miniscule transient accumulation of N_2_O (**Fig S15).** When provided with both N_2_O and NO_3_^-^, all electrons were directed to N_2_O reductase until the external N_2_O was depleted (**Fig S16ABD**). This was expected since the nitrate reductase in **AN** is periplasmic (Nap), and the study of other organisms with Nap has demonstrated that the electron flow to N_2_O reductase (Nos) outcompetes that to Nap when N_2_O is available in excess (Mania et al 2020). **AN** was also apparently *bet hedging*: The electron flow rate declined as the culture switched from oxic to anoxic respiration, and increased exponentially thereafter (**Fig S16C**), which is the typical pattern for a denitrifying organism that performs *bet hedging*. Such organisms express one (or several) of the denitrification enzymes only in a minority of the cells, as demonstrated for *Paracoccus denitrificans* (Lycus et al 2018). The denitrification kinetics indicate that **AN** is *bet hedging* with respect to nitrate reductase (Nap) (**Fig S16, 17**), i.e. that a minority of cells express Nap, while all cells express Nos and Nir, which was corroborated by proteomic analyses which showed very high Nos/Nap protein abundance ratio after transition to anoxic respiration (**Fig S18**). As a consequence, the majority of cells can only reduce (not produce) N_2_O. The *bet hedging* with respect to Nap and the strong competitive edge of Nos versus Nap for electrons explains the cultures capacity to keep N_2_O extremely low when respiring NO_3_^-^ (**Fig S16**), while producing >3 orders of magnitude more N_2_O when provided with NO_2_^-^ (**Fig S17**).

***Azospira* sp. (AS) Fig S19-22.** The phenotype of **AN** was similar to that of **AS**: marginal transient N_2_O accumulation when provided with NO_3_^-^  (**Fig S19**), preferential electron flow to Nos versus Nap (**Fig S21**), but not versus Nir (**Fig S22**), and hence higher N_2_O accumulation, by 2 – 3orders of magnitude, when provided with NO_2_^-^ compared to NO_3_^-^ (**Fig S20**). The electron flow rate during the transition from oxic to anoxic respiration of NO_3_^-^ showed a modest decline in response to oxygen depletion, suggesting that at least 50% of the cells expressed nitrate reductase (**Fig S19**). In contrast, the transition from oxic to anoxic respiration of NO_2_^-^ was “seamless” (i.e. no depression, **Fig S20**), suggesting that all cells expressed nitrite reductase.

***Pseudomonas* *sp*. (PS) Fig S23-25.** The electron flow rates in **PS** during the transition from oxic to anoxic respiration suggested *bet hedging* with respect to the expression of nitrite reductase (**Fig S23 panel B2**), but not nitrate reductase (**Fig S23 panel A2**), and the isolate demonstrated fast depletion of externally provided N_2_O both in the presence of NO_3_^-^ (**Fig S24**) and NO_2_^-^ (**Fig S25**). The gas kinetics indicate that N_2_O reductase in this organism is a very strong sink for electrons, outcompeting both nitrite and nitrate reductase. The steady state N_2_O concentration during anaerobic respiration was low: 50 nM when respiring NO_3_^-^ and 200 nM when respiring NO_2_^-^ (**Fig S25**).

Based on the above phenotypes, **PS** stands out as the most robust N_2_O sink in a complex environment like soil, where NO_2_^-^ inevitably will be produced by other organisms, in response to oxygen depletion.

**Figure S16:** **Denitrification phenotype of *Azonexus* *sp*. (AN) when provided with N_2_O and NO_3_^-^.** The panels A-D show kinetics of gases and NO_2_^-^, calculated electron flow rates and estimation of growth parameters for **AN** grown in gas tight 120 mL vials, initially supplemented with 1 mL O_2_, 1 mL N_2_O and 2mM NO_3_^-^ in 50 mL Sistrom’s succinate medium (headspace volume = 70 mL). The vails were incubated at constant temperature and stirring (20 °C, 700 rpm), and given a dose of 250 µmol N_2_O-N, and 100 µmol NO_3_^-^ after 72 hours. All gases are reported in molar amounts per vial.

Panels A-D show results for a single vial (2 replicate vials gave very similar results, except for a time frameshift with respect to NO_3_^-^ reduction). Panel A: measured O_2_, NO and N_2_O and cumulative N_2_ throughout the incubation (cumulative N_2_ is the measured N_2_ corrected for leakage and loss of N_2_ by sampling, see materials and methods). Inserted panels show measured NO_2_^-^ (nmol vial^-1^) and N_2_O (nmol N vial^-1^). The panel highlights four periods: I, reduction of initial O_2_ and N_2_O; II, reduction of initial NO_3_^-^; III, reduction of the injected 250 µmol N_2_O-N ; IV, subsequent reduction of the remaining NO_3_^-^ (100 µmol NO_3_^-^ was injected together with N_2_O at the beginning of period III). Panel B: N_2_ production rate (***V_N2_***) and N_2_O-reduction rate (***V_N2O_***; this is the rate at which the externally provided N_2_O was reduced). Panel C: Electron flow rates: ***V_eO2_*** is the electron flow rate to terminal oxidases (electron acceptor = O_2_), ***V_eD_*** is the electron flow rate to denitrification reductases (electron acceptors= NO_3_^-^, NO_2_^-^, NO, and N_2_O), ***V_eto_*_t_** = ***V_eO2_*** + ***V_eD_***. The inserted panels show exponential regression of ***V_eO2_*** and ***V_eD_***  against time, thus estimating the aerobic and anaerobic growth rates ($\hat{\mu}_{O2}$ =0.21 h^-1^, $\hat{\mu}_{NO3}$ =0.16 h^-1^). Panel D: Electron flow rates to individual N-reductases (and the sum of all) during the periods III and IV, illustrating the preferential electron flow to Nos (N_2_O→N_2_).

Panel E shows the result of a separate experiment; four replicate vials supplemented with 2 mM NO_3_^-^ and 1 mL O_2_ (no external N_2_O supplied), and initial OD_660nm_ = 0.0064 (inoculum 1 mL, OD_660nm_= 0.32). The panels show exponential regression of N_2_ production rates for each individual vial. The kinetics of O_2_-reduction (not shown) and N_2_-production were used to estimate the fraction of cells expressing Nap (*F_den_*), using the model of Hassan et al (2016). The *F_den_* estimates for the individual vials were 0.12 (vial 1), 0.04 (vial 2), 0.14 (vial 3), and 0.02 (vial 4). The values indicate that **AN** is *bet hedging* with respect to expression of Nap, but that the fraction of cells that express Nap (*F_den_*) varied grossly between vials. Estimated *F_den_* for 9 individual vials (same type of experiment, results not shown) were done, and the *F_den_* estimates ranged from 0.006 to 0.24, average=0.07, stdev=0.08 (result not shown). The NO concentrations during denitrification were invariably low: 0-7 nmol vial^-1^, which is equivalent to 0-5 nM NO in the liquid (1 nmol vial = 0.71 nM in the liquid at the given temperature (20 °C). Likewise, the concentration of N_2_O was extremely low during denitrification: in the vials with NO_3_^-^ only (panel E), the N_2_O-level was 2-4 nmol N_2_O vial^-1^ (=6.4-12.8 nM N_2_O in the liquid, 1 nmol N_2_O-N vial = 3.27 nM N_2_O in the liquid) during the early phase of NO_3_^-^ reduction, increasing gradually to 5-9 nmol N_2_O vial^-1^ during the period with exponentially increasing rates of NO_3_^-^ reduction (results not shown). Such gradual increase in N_2_O concentration is expected for a *bet hedging* organism which expresses N_2_O reductase in all cells and nitrate reductase only in a minority (see Hassan et al 2016).

**Fig S17**: **Denitrification phenotype of *Azonexus* *sp*. (AN) when provided with NO_2_^-^.** The experimental conditions were as for **Fig S16**, but with 1 mM NO_2_^-^ = 50 µmol NO_2_^-^ vial^-1^ (no NO_3_^-^) in the medium. The panel shows measured O_2_, NO and N_2_O and cumulative N_2_ throughout the incubation (cumulative N_2_ is the measured N_2_ corrected for leakage and loss of N_2_ by sampling, see materials and methods), and NO_2_^-^ calculated by N-mass balance (initial NO_2_^-^-N minus N recovered as NO+N_2_O + N_2_-N), all with standard deviation shown as vertical lines (n=2). Peak NO concentrations were ~10 nmol vial^-1^ (~7 nM in the liquid), which is slightly higher than that in the NO_3_^-^ -fed cultures (**Fig S16A**), while the peak N_2_O (20 µmol N_2_O-N vial^-1^) is >3 orders of magnitude higher than during denitrification of NO_3_^-^ (**Fig S16A**). The inserted panel shows the electron flow rates;  ***V_eO2_*** is the electron flow rate to terminal oxidases (electron acceptor = O_2_), ***V_eD_*** is the electron flow rate to denitrification reductases (electron acceptors= NO_2_^-^, NO, and N_2_O), ***V_eto_*_t_** = ***V_eO2_*** + ***V_eD_***. The relatively seamless transition to anoxic respiration (indicated by the only marginal depression in ***V_eto_*_t_** at oxygen depletion) suggests that the majority of cells expressed nitrite reductase.

**
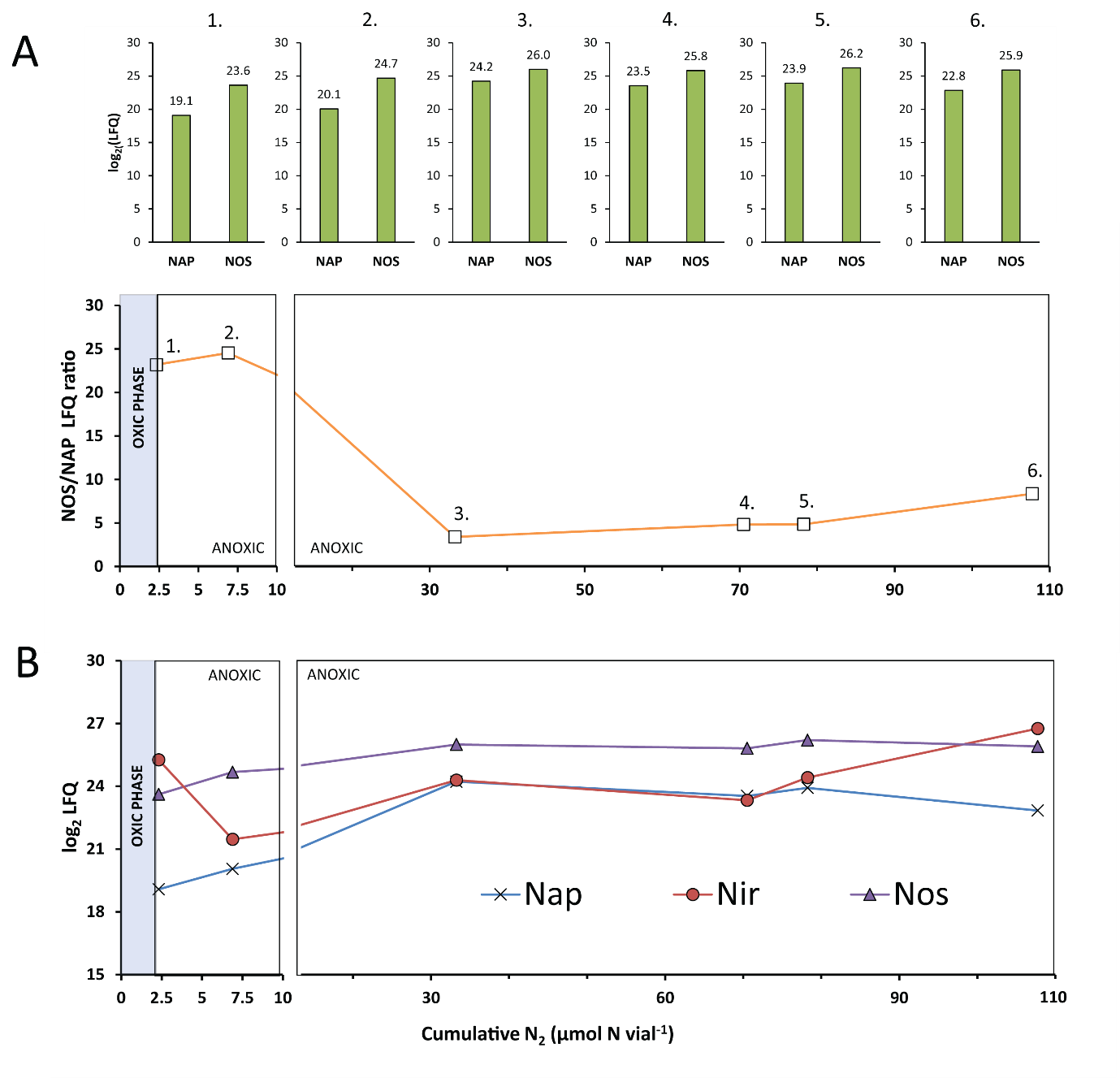
Figure S18**: **Quantification of denitrification reductases for *Azonexus* sp. (AN) by proteomics.** Aerobically grown **AN**- cells were inoculated in replicate vials with 50 mL Sistrom succinate medium supplemented with 2 mM NO_3_^-^ and 1 mL O_2_ (Initial OD_660_ = 0.003 vial^-1^), as for the experiment shown in **Figure** **S16.** Single vials where periodically subjected to destructive sampling and proteomic analysis throughout the incubation (six vials in total, numbered 1- 6). Vial 1 was analyzed at the oxic/anoxic transition (0.7 µM O_2_ in the liquid). Relative LFQ values were corrected for nitrogen reductases with multiple identical subunits (NOS and NIR). Panel A: log_2_(LFQ) values for Nap and Nos (bars) and their ratio, for each sample plotted against the vial-specific cumulative N_2_-N (µmol vial^-1^) at the time of destructive sampling. Panel B: log_2_(LFQ) assigned Nap, Nir and Nos plotted against the cumulative N_2_. Nor was only detected in the final sample. Each figure so split in two parts, with different scales for X-axis to improve visibility of the initial changes.

**Figure S19**: **Denitrification phenotype of *Azospira* *sp*. (AS), provided with NO_3_^-^**. The experimental conditions were as for **Fig S16** but without N_2_O in the headspace. After 50 h, 100 µmol NO_3_^-^ (to a final concentration of 2 mM) was injected. The initial inoculum had OD_660_ = 0.030 (1 mL added to the 50 mL medium in the vials). Error bars indicate standard deviations (n = 3). Panel A: measured O_2_, NO, N_2_O, cumulative N_2_ and NO_3_^-^ calculated by N-mass balance (initial NO_3_^-^-N minus N recovered as NO+N_2_O+N_2_-N). Inserted panels show measured NO_2_^-^ (nmol vial^-1^) and N_2_O (nmol N vial^-1^). All the denitrification intermediates (NO_2_^-^, NO and N_2_O) were extremely low during denitrification. Panel B: Rates of O_2_-consumption (***V_O2_***) and N_2_- production (***V_N2_***). The inserted panels show exponential regression of ***V_O2_*** (oxic phase) and ***V_N2_***, estimating aerobic and anaerobic growth rates (0.23 and 0.20 h^-1^, respectively). Panel C: Calculated electron flow rates: ***V_eO2_*** is the electron flow rate to terminal oxidases (electron acceptor = O_2_), ***V_eD_*** is the electron flow rate to denitrification reductases (electron acceptors= NO_3_^-^, NO_2_^-^, NO, and N_2_O), ***V_eto_*_t_** = ***V_eO2_*** + ***V_eD_***. The dip in electron flow in response to oxygen depletion suggests some *bet hedging* with respect to expression of nitrate reductase (i.e. that ~50% of the cells express nitrate reductase). In contrast, a transition to anoxic respiration with NO_2_^-^ showed no depression in electron flow (**Fig S20, panel C**).

**Figure S19**: **Denitrification phenotype,** ***Azospira* *sp*. (AS), provided with NO_2_^-^**. The experimental conditions were as for **Fig S16**, but with 1 mM NO_2_^-^ (no NO_3_^-^). A dose of NO_3_^-^ (100 µmol NO_3_^-^ vial^-1^) was injected after 50 hours. Error bars indicate standard deviations (n = 3). Panel A: measured O_2_, NO, N_2_O, cumulative N_2_, NO_2_^-^ and NO_3_^-^. NO_2_^-^ and NO_3_^-^ were calculated by N-mass balance (initial amounts minus N recovered as NO+N_2_O+N_2_). Measured NO_2_^-^ is shown as red dots, which are in good agreement with the values based on mass balance. NO was present throughout the entire oxic phase (~20 nmol vial^-1^ = 15 nM NO in the liquid) and peaked to 80 nmol NO vial^-1^ in response to oxygen depletion. The transient N_2_O accumulation during NO_2_^-^ reduction reached 4 µmol N_2_O-N vial^-1^ (12 µM in the liquid), which is 3 order of magnitude higher than N_2_O during denitrification with NO_3_^-^ (**Fig S19**). The onset of NO_3_^-^ reduction after NO_3_^-^ injection was remarkably slow. Panel B: Rates of O_2_-consumption (***V_O2_***), N_2_-production (***V_N2_***). The inserted panels show exponential regression of ***V_O2_*** (oxic phase) and ***V_N2_*** (anoxic phase) estimating aerobic and anaerobic growth rates (0.16 and 0.14 h^-1^, respectively). Panel C: Calculated electron flow rates: ***V_eO2_*** is the electron flow rate to terminal oxidases (electron acceptor = O_2_), ***V_eD_*** is the electron flow rate to denitrification reductases (electron acceptors= NO_2_^-^, NO, and N_2_O), ***V_eto_*_t_** = ***V_eO2_*** + ***V_eD_***. The seamless transition from oxygen- to nitrite-based respiration (no depression in ***V_etot_***) indicate that all cells express nitrite reductase (no *bet hedging*). The response to the subsequent addition of NO_3_^-^ suggests that only a minority of the cells had expressed nitrate reductase, hence, the majority of the cells were unable to utilize nitrate for anaerobic respiration. These cells (without Nap) were likely entrapped in anoxia, without energy to synthesize Nap.

**Figure S21**: **Denitrification phenotype,** ***Azospira* *sp*. (AS), provided with NO_3_^-^** **and N_2_O**. The experimental conditions were as for **Fig S16** (2 mM NO_3_^-^ and 1 mL N_2_O in headspace). 0.3 mL aerobically raised pre culture (OD_660_ = 0.096) was added to 50 mL medium. After 45 hours, a dose of 100 µmol NO_3_^-^ + 160 µmol N_2_O-N (per vial) was injected. The initial inoculum had. The panels show results for a single vial. The replicate vial showed very similar gas kinetics, but with a slight time frameshift. Panel A: measured O_2_, NO and N_2_O and cumulative N_2_ production throughout the incubation. Inserted panels show measured NO_2_^-^ (nmol vial^-1^). Nitrite accumulation was miniscule (inserted panels). Panel B: Rates of O_2_-consumption (***V_O2_***), N_2_-production (***V_N2_***) and N_2_O-reduction (***V_N2O_***). NB: ***V_N2O_*** is the rate of consumption of externally provided N_2_O (positive for N_2_O consumption). The rates of N_2_ production (***V_N2_***) equaled the rates of N_2_O-consumption (***V_N2O_***) during depletion of exogenous N_2_O. This shows that during the transition from oxic to anoxic conditions, N_2_O was converted stoichiometrically to N_2_, while nitrate was only reduced after depletion of N_2_O. The inserted panels show exponential regression of ***V_O2_*** (oxic phase) and ***V_N2_*** (anoxic phase) estimating aerobic and anaerobic growth rates (0.21 and 0.20 h^-1^, respectively).

**Figure S22**: **Denitrification phenotype,** ***Azospira sp*. (AS) provided with NO_2_^-^ and N_2_O**. Experimental condition as for **Fig S16**, but with 1 mL N_2_O and 1 mM NO_2_^-^. After 45 h, a dose of 100 µmol NO_3_^-^ + 160 µmol N_2_O-N (per vial) was injected. The panels show the average of two replicate vials. Panel A: measured O_2_, NO, N_2_O, N_2_ (cumulative) and NO_2_^-^. Measured NO_2_^-^ during the depletion of externally supplied N_2_O is shown in the main panel, while the miniscule NO_2_^-^ measured after injection of NO_3_^-^ is shown in the inserted panel. During the transition from oxic to anoxic conditions NO_2_^-^ - and N_2_O- was reduced concomitantly. Following addition of 100 µmol NO_3_^-^ and 160 µmol N_2_O-N *Azospira* sp. AS quickly reduced exogenous supplied N_2_O, but the immediate NO_3_^-^ reduction rates was miniscule and gradually increasing. Panel B: rates of O_2_- and N_2_O-reduction, and N_2_ production. During the depletion of exogenous N_2_O with NO_2_^-^ present (time span 22-30h), N_2_ production rates clearly exceeded the rates of N_2_ production, reflecting concomitant reduction of NO_2_^-^ and the exogenous N_2_O. During the depletion of exogenous N_2_O in the presence of NO_3_^-^ (time span 42-47 h), the rate of N_2_ production did not exceed the rate of N_2_O reduction, hence no NO_3_-reduction took place. Inserted panels: exponential regression (against time) of the rates of O_2_-reduction (oxic phase) and N_2_-production.

**Figure S23**: **Denitrification phenotype of** ***Pseudomonas* *sp*. (PS), provided with NO_3_^-^ or NO_2_^-^**. The experimental conditions were as for **Fig S16**; vials, with 50 mL medium were supplemented with 1 mL O_2_ and 2mM NO_3_^-^ (=100 µmol NO_3_^-^ vial^-1^ (panels A1&2, n = 3 replicate vials) or 1 mM NO_2_^-^ (Panel B1&2, n = 2 replicate vials). The vials were inoculated with 1 mL of a culture with OD_660nm_ = 0.06. Panel A1: measured gases and nitrogen NO_2_^-^ in vials with 2 mM NO_3_^-^. The panel also shows NO_3_^-^ calculated by N mass balance (initial amount of NO_3_^-^- N minus N recovered as (NO_2_^-^+NO+N_2_O+N_2_)-N). The figure shows transient nitrite accumulation to 75 µmol vial^-1^, while the NO and N_2_O remained very low (50 nmol NO vial^-1^ ~35 nM in the liquid, 70 nmol N_2_O-N vial^-1^ ~ 230 nM N_2_O in the liquid). Panel A2 shows the calculated electron flow rates to O_2_ (***V_eO2_***) and to denitrification reductases (***V_eD_***), and the total electron flow rate (***V_etot_***=***V_eO2_*+*V_eD_***). The seamless transition from aerobic respiration to respiration by NO_3_^-^ -reduction (marginal reduction of **V_etot_**) suggests that all cells express nitrate reductase. The reduction of **V_etot_** in response to NO_3_^-^ depletion suggest that only a fraction of the cells express nitrite reductase. Panel A3: the vials (Panel A1&2) were given a dose of 100 µmol NO_3_^-^ and 100 µmol N_2_O after 49 hours. The kinetics reveal a strong preference for N_2_O over NO_3_^-^.

Panel B1: Measured gases in vials with 1 mM NO_2_^-^. The panel also shows NO_2_^-^ calculated by N mass balance (initial NO_2_^-^ minus N recovered as (NO+N_2_O+N_2_)-N minus that. The figure shows that transient accumulation of intermediates reached 200 nmol NO vial (~140 nM in the liquid) and 190 nmol N_2_O-N vial^-1^ (~620 nM in the liquid). Panel B2: calculated electron flow rates to O_2_ (***V_eO2_***), to denitrification reductases (***V_eD_***), and the total electron flow (***V_etot_***). The dip in the electron flow after the transition from aerobic to anaerobic respiration suggests that only a fraction of cells express nitrite reductase. Panel B3: the vials (B1&2) were given a dose of NO_3_ and N_2_O after 49 hours.

**Figure S24:**  **Denitrification phenotype of *Pseudomonas* *sp*. (PS), provided with NO_3_^-^** **and N_2_O**. Experimental conditions as in **Figure 16** (N_2_O in headspace, 2 mM NO_3_^-^ in the medium). Panel A shows measured O_2_, NO, N_2_O, N_2_. Panel B shows rates of O_2_-consumption (***V_O2_***), N_2_O consumption (***V_N2O_***) and N_2_-production (***V_N2_***). Error bars: standard deviation, n = 2. The inserted panel shows estimated aerobic growth rate (exponential regression of O_2_ reduction rate against time). The electron flow rates to the individual steps could not be calculated in this experiment because NO_2_^-^ was not measured.

**Figure S25**: ***Pseudomonas* *sp*. PS, elucidating the preference for N_2_O versus NO_2_^-^ and NO_3_**^-^. To assess the preferential reduction of N_2_O versus NO_2_^-^ and NO_3_^-^, we set up an experiment with 5 vials with 2mM NO_2_^-^ and 1 mL N_2_O (as **Fig S16**) and monitored the gas kinetics. After depletion of all electron acceptors (N_2_O-N + NO_2_-N recovered as N_2_, Panel A1 and A2), the experiment was continued by injecting more electron acceptors: two of the vials received 100 µmol NO_3_^-^ (2 mM NO_3_^-^) + 1 mL N_2_O (Panels B1 and B2), while three vials received 100 µmol NO_2_^-^ + 1 mL N_2_O (Panel C1 and C2). The time of injections are indicated by black arrows (panel B1&C1). Top panels (A1-C1) show measured gases (molar amounts per vial), with inserted panels showing the N_2_O concentration in the liquid (nM, log scale), illustrating steady state N_2_O concentrations during respiration based on nitrogen oxyanion-reduction alone, i.e. after depletion of the externally provided N_2_O (these steady state concentrations are indicated by dashed red lines). These steady state concentrations were ~200 nM when respiring NO_2_^-^ (panel A1 and C1), and ~50 nM when respiring NO_3_^-^ (panel B1), and again 200 nM when respiring NO_2_^-^. The lower panels (A2-C2) show calculated rates of O_2_-consumption (*V_O2_*), N_2_O-depletion (*V_N2O_* = the rate at which exogenous N_2_O was depleted), and N_2_-production (*V_N2_*). Calculated electron flow rates are shown in the inserted panels. The insert in panel A2 shows the electron flow to terminal oxidases (marked O_2_), and to the denitrification reductases Nir and Nos and the total (the electron flow to Nor is practically identical with that to Nir since only nanomolar amounts of NO accumulated). The inserts in panel B2 and C2 show electron flow to Nos and Nir only (no oxygen was present in these vials). The electron flow to Nar could not be estimated for (Panel B2) because NO_2_^-^ was not measured.

The initial incubation (Panels A1&A2) demonstrates a strong preference for external N_2_O versus NO_2_^-^, although the electron flow to Nir increased gradually as the concentration of exogenous N_2_O declined. In response to a second dose of N_2_O + NO_2_ (panel C1 and C2), we see the same preference for N_2_O versus NO_2_^-^.

**G. Aerobic growth in sterilized digestate, and the effect of the enriched digestate on N_2_O emissions**

The cultures where grown in pre-aerated digestate and supplied with O_2_ as the terminal electron acceptor prior to inoculation in soil (Soil incubations shown in Figure 5 (main paper) and Figure S28 and S29). The pre-aeration, done before inoculation of isolated cultures by blowing air through the sterile digestate suspension for 72 hours, was necessary to secure near-complete abiotic oxidation of the Fe^2+^ in the digestate before inoculation of the cultures (FeCl_3_ is used at a precipitation chemical at the WWTP, se materials and methods). Fe^2+^ would otherwise obscure the measurements of O_2_ consumption, and possibly inhibit aerobic respiration due to formation of reactive oxygen species (Winterbourn 1995).

**Figure S26**: **Aerobic growth in autoclaved digestate.** 1 mL cultures of PS, AS and AN, grown oxicly (air) in stirred (700 rpm) Sistrom medium at 20 °C, were inoculated at 20 °C in closed 120 mL stirred vials (600 rpm) containing 50 mL autoclaved, pre-aerated and pH-adjusted (pH=7.5) digestate and monitored for gas concentrations of O_2_ in the headspace. Inoculum OD_660nm_ in digestate was 0.26, 0.12 and 0.30 for cultures PS, AS and AN, respectively. The vials were helium flushed and 5 mL O_2_ was added to the headspace before inoculation of the sioaltes. Panels A to D shows calculated liquid concentration of O_2_ (µM) and rate of oxygen consumption on the primary y-axis (log_10_ scaled), and cumulative O_2_ consumed (µmol mL^-1^) throughout the incubation for the cultures PS (two replicates), AS (two replicates), AN (single vial) and a control vial containing digestate only (one vial), respectively. Error bars = standard deviation. Fat arrows represent replenishing of oxygen using a syringe piercing the rubber septum of the vials. Liquid concentration of oxygen was not calculable for the timepoint following O_2_ addition and is therefore removed. Culture PS (Panel A) consumed significantly more O_2_ in digestate compared to cultures AS (Panel B) and AN (Panel C). We therefore added a 1 mL of a carbon mix to vials of AN and PS (point of addition is indicated in panel B and C). The carbon mix contained 0.5 mM glutamate, 0.5 acetate, 0.5 mM puryvate and 0.5 mM ethanol dissolved in sterile water and pH adjusted to 7. The cumulative end point O_2_ consumption per mL of digestate suspension is shown in Panel A – D. Assuming growth yields on oxygen to 1.5E14 cells mol^-1^ O_2_, as determined for *Paracoccus denitrificans* (Bergaust et al 2010, 2012), and correcting for abiotic oxygen consumption in the control (Panel D) a cell density of 3.2E09, 3.3E09 and 2.4E09 mL^-1^ for *Pseudomonas sp*. PS, *Azospira* *sp*. AS and *Azonexus* *sp*. AN, respectively, was estimated.

**Figure S27. Aerobic and anaerobic growth of isolates in autoclaved digestates.** Panel A shows a compilation of the cumulated O_2_ consumption during aerobic incubations, as shown in more detail in Fig S26. The timepoint of adding a carbon substrate cocktail to AS and AN (see Fig. S26) is indicated by arrows. (standard deviation is not included). Panel B shows the cumulated N_2_ production in identical corresponding vials, but with a He + N_2_O atmosphere. No carbon substrates were added to these vials. Error bars = standard deviation (n = 3).

**Figure S28: Incubation of digestates treated in various ways with soil with pH=5.5.** Panel A: kinetics of O_2_, NO, N_2_O and N_2_ throughout the incubation of soils amended with the various materials (one panel for each amendment) given as molar amounts per vial. The panels show average values (n=2). The initial oxygen (~20 µmol vial^-1^ corresponding to ~0.5 vol% in the headspace) was depleted within the first 20 hours for soils amended with digestates, while soil alone (lower panel) took around 100 hours to deplete O_2_. This is due to the boost in respiration that occurs when the carbon-rich digestate is added to the soil. The amounts of O_2_, NO and N_2_O are as measured, while “Cumulative N_2_” denotes the measured N_2_ that is corrected for leakage and losses by sampling (see Molstad et al 2007). The N_2_ and N_2_O kinetics were used to calculate the N_2_O index (***I_N2O_***), which is the area under the N_2_O-curve divided by the sum of the areas under the N_2_O and N_2_ -curves for a specific time span. ***I_N2O_*** values are shown in **Fig 5** (main paper) and provide a proxy for the propensity of the system to emit N_2_O. Panel B: peak (maximum) amounts of NO and N_2_O (results for single vials, 2 vials per condition; average value indicated). NO is shown as nM in the liquid phase (equilibrium concentrations with measured NO in headspace), while N_2_O is shown as µmol N_2_O-N vial^-1^.

**Figure S29: Incubation of isolates and N_2_O enriched digestate in soil with pH=6.6.** Panel A: kinetics of O_2_, NO, N_2_O and N_2_ throughout the incubation of soils amended with the various materials (one panel for each amendment). Average values shown (n=2). The initial oxygen (~20 µmol vial^-1^, ~0.5 vol% in the headspace) was depleted within the first 20 hours for soils amended with digestates, while soil alone (lower panel) took around 100 hours to deplete O_2_. The amounts of O_2_, NO and N_2_O are as measured, while “Cumulative N_2_” denotes the measured N_2_ that is corrected for leakage and losses by sampling (see Molstad et al 2007). The N_2_ and N_2_O kinetics were used to calculate the N_2_O index (***I_N2O_***), which is the area under the N_2_O- curve divided by the area under the N_2_O+N_2_ -curve for a specific time span. ***I_N2O_*** values are shown in **Fig 5** (main paper) and is a proxy for the propensity of denitrification to emit N_2_O. Panel B: peak (maximum) amounts of NO and N_2_O (results for single vials). NO is shown as nM in the liquid phase (equilibrium concentrations with measured NO in headspace), while N_2_O is shown as µmol N_2_O- N vial^-1^.

**Figure S30: Potential methanogenesis in soil amended with digestates.** The panels show the rate of N_2_-production (***V_N2_***) and rate of methane production (**V_CH4_**) during incubations of soils amended with digestates (see **Figures S28 & S29**). Panels A1-A3 show results for soil with pH 5.5, and panels B1-B3 show results for soil with pH 6.6. The three panels for each soil show the results for 1) soils amended with digestate enriched with N_2_O-reducing bacteria “N_2_O-enrichment” (Fig 2B, main paper) 2) soils amended with “live digestate” (i.e. digestate taken directly from the anaerobic digester and 3) soils amended with digestate that had been heated to 70 ^o^C for 2 hours. The results demonstrate that the methanogenic consortium of the digestate is alive and active in soil, but evidently suppressed by denitrification: once the nitrogen containing electron acceptors are depleted (V_N2_ approaches zero), methanogenesis resumed. This did not happen in soils amended with the digestate that had been heated to 70 ^o^C and thus likely did no longer contain live methanogenic bacteria.

**Figure S31: Storage experiment.** To test the short term survival of the N_2_O-scavenging capacity of N_2_O reducers, we added 3 mL freshly sampled live digestate or 3 mL N_2_O enriched digestate from the enrichment shown in Figure 2B in main paper to 10 g soil (pH 6.6) in 6 vials of which 3 were incubated anaerobically immediately for measurement of gas kinetics (“Live dig t = 0h” and “N2O enr. t = 0h”), and 3 were stored aerobically (open vials) for 70 hours in soil (“Live dig in soil t = 70h” and “N2O enr. in soil t = 70h”) before being incubated anaerobically in the automated incubation and gas analysis system. In parallel, we also stored 50 mL of the freshly sampled digestate and 50 mL enrichment culture aerobically in open vials for 70 hours from which 3 mL digestate was then applied to 10 g soil (pH 6.6) (“N2O enr. t = 70 h” and “Live dig t = 70h”). Aerobic storage of digestates and digestate amended soil was conducted at 20 °C. Monitoring of gas kinetics was conducted at 20 °C. All treatments were supplemented with 50 µmol NO_3_^-^ (50 µL 1 M KNO_3_) just before gas analysis in.

Panel A shows the N_2_O kinetics for all six soil treatments; the inserted panel shows the results for the two treatments with so low N_2_O levels that they would be invisible in the main panel (n=3 for all treatments, standard deviations are shown as vertical bars). Panel B shows maximum NO, N_2_O and the N_2_O index for 40 % recovery of NO_3_^-^ as N-gases (***I_N2O40%_***). NO is shown as nM in the liquid phase (equilibrium concentrations with measured NO in headspace), while N_2_O is shown as µmol N_2_O- N vial^-1^. The results show that within a time frame of 70 h under aerobic conditions, be it as intact enrichment or after amendment to soil, the capacity to reduce the N_2_O/N_2_ product ratio of denitrification in soil is sustained.

**References**

Anja K, Schink B, Müller N (2019) Energy-conserving enzyme systems active during syntrophic acetate oxidation in the thermophilic bacterium *Thermacetogenium phaeum*. Frontiers in Microbiology 10:2785. DOI:10.3389/fmicb.2019.02785

Bakken LR (2021) Spreadsheet for gas kinetics in batch cultures: KINCALC. DOI:10.13140/RG.2.2.19802.36809

Bergaust L, Mao Y, Bakken LR, Frostegård Å (2010) Denitrification response patterns during the transition to anoxic respiration and posttranscriptional effects of suboptimal pH on nitrogen oxide reductase in *Paracoccus denitrificans*. Applied and Environmental Microbiology 76:6387-6396. DOI:10.1128/AEM.00608-10

Botheju D, Bakke R (2011) Oxygen Effects in Anaerobic Digestion – A Review. The Open Waste Management Journal 4:1-19. DOI:10.2174/1876400201104010001

Cheng C, Shen X, Xie H, Hu Z, Pavlostathis SG, Zhang J (2019) Coupled methane and nitrous oxide biotransformation in freshwater wetland sediment microcosms. Science of the Total Environment 648:916-922. DOI:10.1016/j.scitotenv.2018.08.185

Elsgaard L, Olsen AB, Petersen SO (2016) Temperature response of methane production in liquid manures and co-digestates. Science of the Total Environment 539:78-84. DOI:10.1016/j.scitotenv.2015.07.145

Dyksma S, Jansen L, Gallert C (2020) Syntrophic acetate oxidation replaces acetoclastic methanogenesis during thermophilic digestion of biowaste. Microbiome 8:1-14. DOI:10.1186/s40168-020-00862-5

Hassan J, Qu Z, Bergaust L, Bakken LR (2016) Transient accumulation of NO_2_^-^ and N_2_O during denitrification explained by assuming cell diversification by stochastic transcription of denitrification genes. PLoS Computational Biology 12:e1004621. DOI:10.1371/journal.pcbi.1004621

Hein S, Witt S, Simon J (2017) Clade II nitrous oxide respiration of *Wolinella succinogenes* depends on the NosG, ‐C1, ‐C2, ‐H electron transport module, NosB and a Rieske/cytochrome *bc* complex. Environmental Microbiology 19:4913-4925. DOI:10.1111/1462-2920.13935

Jonassen KR, Ormåsen I, Duffner C, Hvidsten TR, Frostegård Å, Bakken LR, Vick SHW (2021) A novel dual enrichment strategy provides soil- and digestate-competent N_2_O-respiring bacteria for mitigating climate forcing in agriculture. BiorXiv 2021 doi: https://doi.org/10.1101/2021.05.11.443593

Lycus P, Soriana-Laguna MJ, Kjos M, Richardson DJ, Gates AJ, Milligan DA, Frostegård Å, Bergaust L, Bakken LR (2018) A bet-hedging strategy for denitrifying bacteria curtails their release of N_2_O. Proceedings of the National Academy of Sciences USA 115:11820-11825. [DOI:10.1073/pnas.1805000115](https://doi.org/10.1073/pnas.1805000115)

Mania D, Wolily K, Degefu T, Frostegård Å (2020) A common mechanism for efficient N_2_O reduction in diverse isolates of nodule-forming bradyrhizobia. Environmental Microbiology 22:17-31. DOI:10.1111/1462-2920.14731

Molstad L, Dörsch P, Bakken L (2007) Robotized incubation system for monitoring gases (O_2_, NO, N_2_O, N_2_) in denitrifying cultures. Journal of Microbiological Methods 71:202-211. DOI:10.1016/j.mimet.2007.08.011

Mosbæk F, Kjeldal H, Mulat DG, Albertsen M, Ward AJ, Feilberg A, Nielsen JL (2016) Identification of syntrophic acetate-oxidizing bacteria in anaerobic digesters by combined protein-based stable isotope probing and metagenomics. The ISME Journal 10:2405-2418. DOI:10.1038/ismej.2016.39

Swanson MA, Usselman RJ, Frerman FE, Eaton GR, Eaton SS (2008) The iron− sulfur cluster of electron transfer flavoprotein−ubiquinone oxidoreductase is the electron acceptor for electron transfer flavoprotein. Biochemistry 47:8894-8901. DOI:10.1021/bi800507p

Vaccaro BJ, Thorgersen MP, Lancaster WA, Price MN, Wetmore KM, Poole FL, Deutschbauer A, Arkin AP, Adams MW (2016) Determining roles of accessory genes in denitrification by mutant fitness analyses. Applied and Environmental Microbiology 82:51-61. DOI:10.1128/AEM.02602-15

Valenzuela EI, Padilla-Loma C, Gómez-Hernández N, López-Lozano NE, Casas-Flores S, Cervantes FJ (2020) Humic substances mediate anaerobic methane oxidation linked to nitrous oxide reduction in wetland sediments. Frontiers in Microbiology 11:587. DOI:10.3389/fmicb.2020.00587

Wunsch P, Zumft WG (2005) Functional domains of NosR, a novel transmembrane iron-sulfur flavoprotein necessary for nitrous oxide respiration. Journal of Bacteriology 187:1992–2001. DOI:10.1128/JB.187.6.1992-2001.2005

Zhang L, Trncik C, Andrade SL, Einsle O (2017) The flavinyl transferase ApbE of *Pseudomonas stutzeri* matures the NosR protein required for nitrous oxide reduction. Biochimica et Biophysica Acta 1858:95–10. DOI:10.1016/j.bbabio.2016.11.008
